# Computed Tomography Demonstration of the Production and Distribution of Oxygen Gas Following Intratumoral Injection of a New Radiosensitizer (KORTUC) for Patients with Breast Cancer—Is Intratumoral Injection Not an Ideal Approach to Solve the Major Problem of Tumor Hypoxia in Radiotherapy?

**DOI:** 10.3390/cancers8040043

**Published:** 2016-04-01

**Authors:** Naoya Hayashi, Yasuhiro Ogawa, Kei Kubota, Kazuhiro Okino, Ryo Akima, Shiho Morita-Tokuhiro, Akira Tsuzuki, Shin Yaogawa, Akihito Nishioka, Mitsuhiko Miyamura

**Affiliations:** 1 Division of Radiology, Medical School Hospital, Kochi University, Nankoku, Kochi 783-8505, Japan; jm-n.hayashi@kochi-u.ac.jp (N.H.); jm-okinok@kochi-u.ac.jp (K.O.); jm-akima.r@kochi-u.ac.jp (R.A.); jm-tokuhiros@kochi-u.ac.jp (S.M.-T.); jm-tsuzukia@kochi-u.ac.jp (A.T.); jm-yaogawa@kochi-u.ac.jp (S.Y.); 2 Hyogo Prefectural Kakogawa Medical Center, Kakogawa, Hyogo 675-8555, Japan; 3 Department of Diagnostic Radiology & Radiation Oncology, Medical School Hospital, Kochi University, Nankoku, Kochi 783-8505, Japan; kubotak@kochi-u.ac.jp (K.K.); nishiokaa@kochi-u.ac.jp (A.N.); 4 Department of Pharmacy, Medical School Hospital, Kochi University, Nankoku, Kochi 783-8505, Japan; miyamus@kochi-u.ac.jp

**Keywords:** hydrogen peroxide, radiosensitizer, sodium hyaluronate, radiotherapy, KORTUC, tumor hypoxia, radiation therapy

## Abstract

We previously developed a new enzyme-targeting radiosensitization treatment named Kochi Oxydol-Radiation Therapy for Unresectable Carcinomas, Type II (KORTUC II), which contains hydrogen peroxide and sodium hyaluronate for injection into various types of tumors. For breast cancer treatment, the radiosensitization agent was injected into the tumor tissue twice a week under ultrasonographic guidance, immediately prior to each administration of radiation therapy. At approximately three hours after the second or third injection, computed tomography (CT) was performed to confirm the production and distribution of oxygen gas generated from the KORTUC radiosensitization agent by catalysis of peroxidases contained mainly in tumor tissue. The purpose of this study was to demonstrate that tumor hypoxia could be overcome by such a procedure and to evaluate the method of intratumoral injection in terms of confirming oxygen distribution in the target tumor tissue and around the tumor to be visualized on dedicated CT imaging. Three-dimensional reconstructed maximum intensity projection imaging of contrast-enhanced breast magnetic resonance imaging was used to compare the position of the tumor and that of the generated oxygen. Distributed oxygen gas was confirmed in the tumor tissue and around it in all 10 patients examined in the study. A region of oxygen gas was measured as an average value of −457.2 Hounsfield units (HU) as a region of interest. A slightly increased HU value compared to the density of air or oxygen was considered due to the presence of tumor tissue in the low-density area on 5-mm-thick reconstructed CT imaging. The results of this study showed that intratumoral oxygen was successfully produced by intratumoral KORTUC injection under ultrasonographic guidance, and that tumor hypoxia, which is considered a main cause of radioresistance in currently used Linac (linear accelerator) radiation therapy for malignant neoplasms, could be resolved by this method.

## 1. Introduction

Low linear energy transfer (LET) radiation, such as X-rays and electron beams from a linear accelerator (Linac), is mainly used for clinical radiotherapy worldwide. However, the therapeutic effect of radiotherapy using a Linac for relatively large tumors of more than several centimeters in diameter decreases to one-third of that of smaller tumors due to tumor hypoxia and the abundance of anti-oxidative enzymes, such as many kinds of peroxidases and catalase [1,2].

Therefore, we recently developed a new enzyme-targeting radiosensitization treatment, KORTUC (Kochi Oxydol-Radiation Therapy for Unresectable Carcinomas) using hydrogen peroxide and sodium hyaluronate for intratumoral injection [3,4,5,6,7,8,9], and the safety and effectiveness of the treatment have been demonstrated mainly for patients with locally-advanced neoplasms [10,11,12,13,14,15,16,17,18,19].

To confirm oxygen production and evaluate the distribution of oxygen gas, CT examinations were performed approximately 3 h after the second or third radiosensitization agent injection for 10 patients for whose breast cancer it seemed to be relatively difficult to obtain an even distribution of oxygen gas throughout the target tumor guided by ultrasonographic studies alone.

The purpose of this study was to demonstrate oxygen gas production in the tumor, which would demonstrate a possible ideal approach for solving the major problem of tumor hypoxia [20] for low LET radiotherapy, and to evaluate the method for KORTUC intratumoral injection under ultrasonographic guidance.

## 2. Results

In all 10 patients shown in Table 1, production and distribution of oxygen gas were confirmed in and around the tumor tissue.

Low-density areas due to oxygen gas production were measured as an average of −457.2 Hounsfield units (HU) in the ipsilateral breast tumor on computed tomography (CT) images.

**Figure 1:**
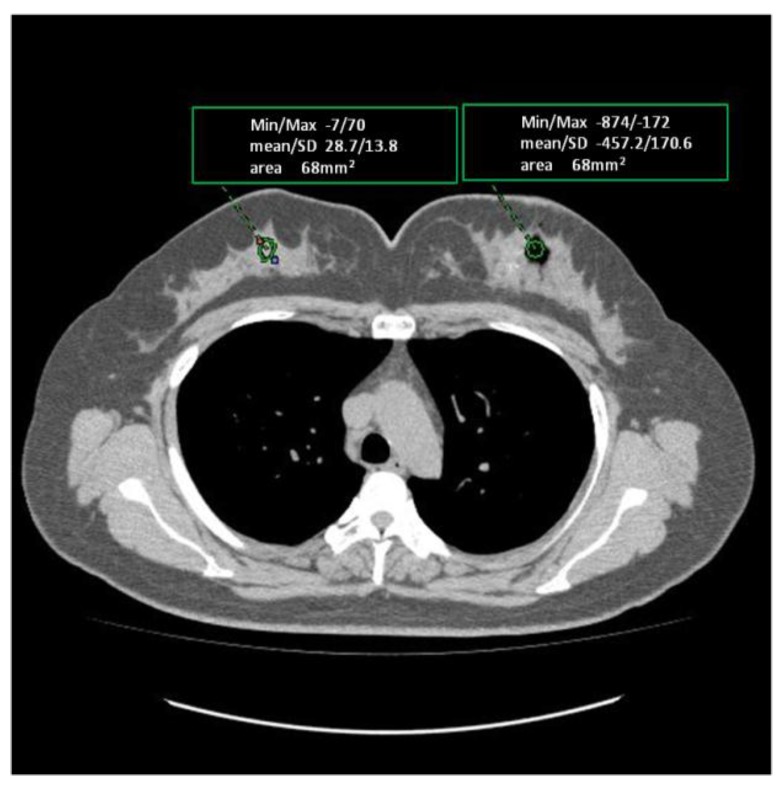
Comparison of the Hounsfield unit values between a region of oxygen gas production in the target tumor in the ipsilateral breast and the normal mammary gland tissue in the contralateral breast.

In the contralateral breast, average density of the normal mammary gland tissue was 28.7 HU on CT images. These data are shown in Figure 1.

**Figure 2:**
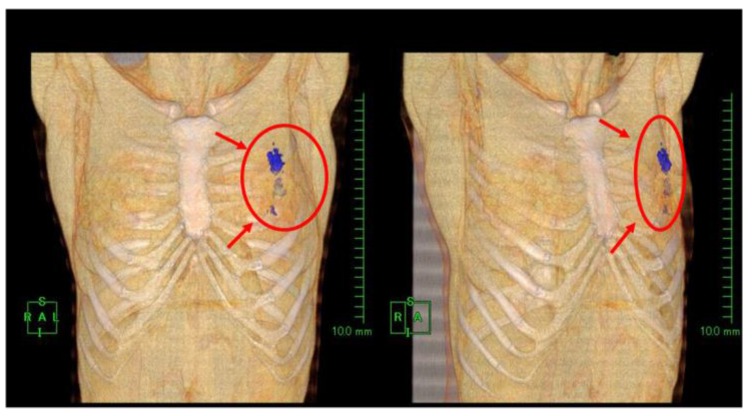
Case 2: 3D reconstructed computed tomography (CT) images taken at approximately 3 h after injection of the KORTUC agent. Oxygen gas distributions (blue color) are identified in the left breast tissue, including the breast cancer.

**Figure 3:**
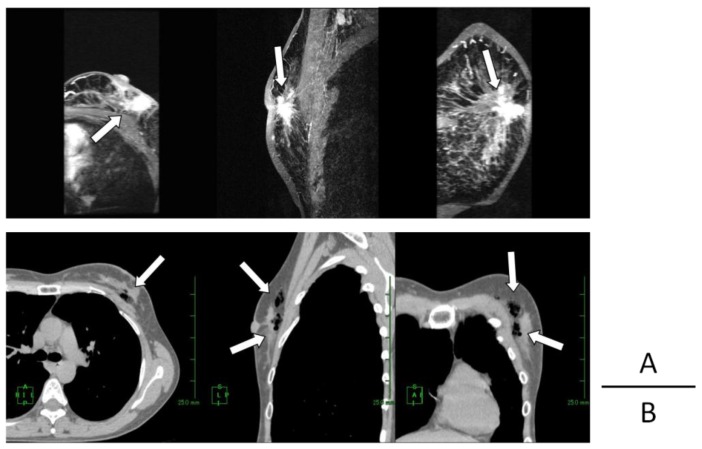
Case 2: Comparison of the position of tumor (arrows in **A**) and that of generated oxygen gas (arrows in **B**) (**A**) 3D-MIP (maximam intensity projection) images of pre-treatment contrast-enhanced (CE) breast MRI (**B**) 2D reconstructed breast CT images taken approximately 3 h after KORTUC radiosensitization agent injection.

**Figure 4:**
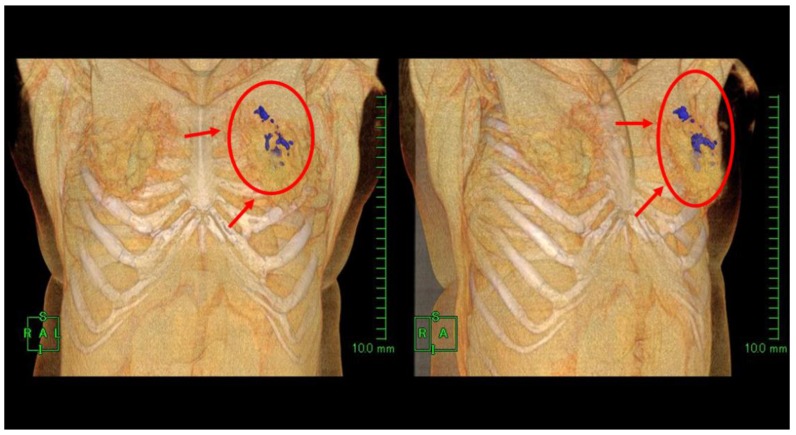
Case 6: 3D reconstructed CT images taken approximately 3 h following intratumoral KORTUC injection. Oxygen gas distributions (blue color) are identified in the left breast tissue, including the breast cancer.

**Figure 5:**
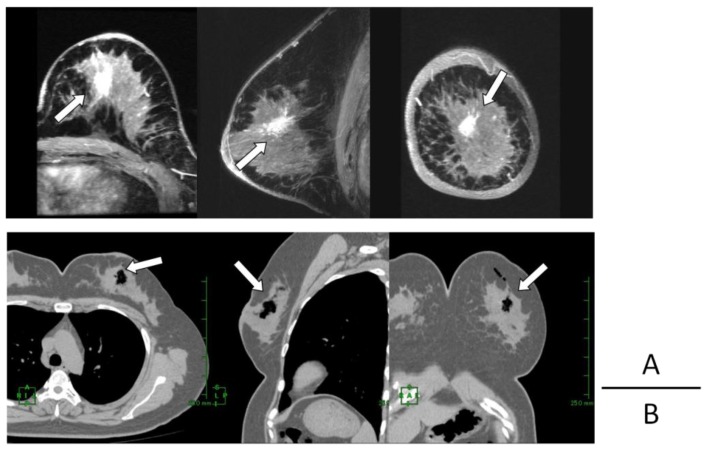
Case 6: Comparison of the position of tumor (arrows in **A**) and of generated oxygen gas (arrows in **B**) (**A**) 3D-MIP images of pre-treatment CE breast MRI (**B**) 2D reconstructed breast CT images taken approximately 3 h after KORTUC radiosensitization agent injection.

Examples of production and distribution of oxygen in and around the target breast tumor tissue on CT images, along with comparisons with contrast-enhanced (CE) breast MRI, are shown in Figure 2, Figure 3, Figure 4 and Figure 5.

## 3. Discussion

The HU values of each tissue on CT examination are shown in Table 2.

Based on the CT findings of the low-density area showing a minimum HU value of −874 HU, one can conclude that the object seen on the CT examination is gas, such as air or oxygen gas. Regarding the kind of gas, our previous experimental results confirmed that the gas generated from the KORTUC radiosensitization agent is oxygen [3,4,5,8].

Therefore, we concluded that the gas seen following KORTUC intratumoral injection on CT imaging was oxygen gas.

Table 2 also indicates that the HU value of air or oxygen gas is −1000 HU, and the average of low-density area on CT examination was −457.2 HU in the present study. It was considered that a slightly increased value of −457.2 HU, compared to that of air or oxygen gas, was due to the presence of tumor tissue in the region on 5-mm-thick reconstructed CT images.

It has been shown that approximately two thirds of the therapeutic effect of X-rays and electrons from a Linac (linear accelerator) are due to the radical reactions they cause. In the cytoplasm, they are mainly brought about by the formation of radicals, such as hydroxyl radicals, by radiation degradation of the intrabody and/or intracellular water molecules. Therefore, in the circumstances of both tumor hypoxia and an abundance of anti-oxidative enzymes, such as many kinds of peroxidases and catalase, in tumor tissues, the therapeutic effects of X-rays and electrons are decreased to one-third of their ideal efficacy [1,2].

**Figure 6:**
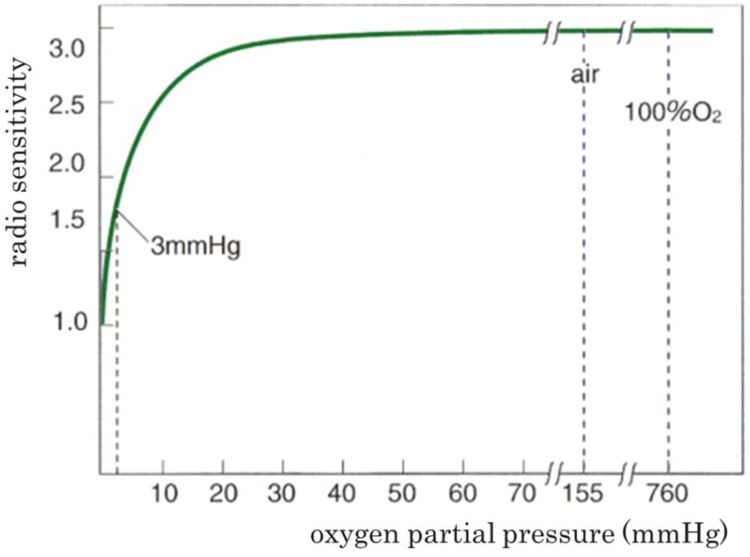
The relationship between oxygen partial pressure and the relative radio-sensitivity.

In this study, the production of oxygen and oxygen distribution were confirmed in and around tumor tissue in all 10 patients examined. However, it was not confirmed that the generated oxygen was enough to yield ideal circumstances for low-LET radiation such as X-rays and electrons in terms of exerting a full therapeutic effect. Regarding the problem mentioned above, Tokuhiro *et al*. showed in their experimental studies that oxygen partial pressure in the central part of tumor tissue was approximately 1000 mmHg at one hour and approximately 90 mmHg at 24 h following KORTUC injection [8]. As the relationship between oxygen partial pressure and relative value of radiosensitivity shows in Figure 6, intratumoral oxygen partial pressure of less than 20–30 mmHg causes deterioration of radiosensitivity [1].

The oxygen partial pressure value of 90 mmHg at 24 h following intratumoral injection of the KORTUC agent shown in the experimental study still far exceeds this value of 20–30 mmHg. Therefore, the generated oxygen can be enough to obtain ideal circumstances for X-rays and electrons to fully exert their therapeutic effects against individual tumors if a sufficient volume of the KORTUC agent is inserted into the tumor tissue.

Based on the results mentioned above, intratumoral injection of the KORTUC agent twice a week can provide full reoxygenation of tumor hypoxia for radiation therapy of five times per week. Basically, the present CT demonstration of the production and distribution of oxygen gas throughout the tumor tissue following intratumoral injection of the KORTUC agent implies that tumor hypoxia, which for a long time has been considered the main cause of radioresistance to X-rays and electrons, can be easily and safely converted to a hyperoxic state with the KORTUC method.

## 4. Patients and Methods

A new radiosensitizer named KORTUC containing 0.5% *w/v* hydrogen peroxide and 0.83% *w/v* sodium hyaluronate (a CD44 ligand) has been developed for intratumoral injection into various radioresistant tumors. This new method, named KORTUC II, was approved by our local ethics committee at Kochi University Medical School for the treatment of breast cancer, metastatic lymph nodes, malignant melanoma, and soft tissue sarcoma, *etc.*

Between April 2011 and March 2013, 10 female patients with breast cancer with tumors for which successful intratumoral injection of the KORTUC agent seemed to be relatively difficult under ultrasonographic guidance alone underwent CT examinations to ascertain production and distribution of oxygen gas in the target tumor tissue at approximately three hours following intratumoral injection. Each patient provided her written, informed consent before participation in the study.

Patient data are summarized in Table 1.

For radiation therapy, treatment planning was performed using Pinnacle^3^, and hypofraction radiotherapy was administered using a tangential fields approach, including an ipsilateral axillary region and the field-in-field method. The energy level was 4 MV, and the total dose was 44 Gy, administered as 2.75 Gy/fraction for patients with early-stages breast cancer [21]. An electron boost of 3 Gy was added three times following the 14th, 15th, and 16th administrations of X-ray irradiation, using an electron beam of appropriate energy for each individual patient. For patients with locally-advanced breast cancer, the total dose was 49.5 Gy administered as 2.75 Gy/fraction.

A maximum of 9 mL (usually 3 mL for tumors of less than approximately 3 cm in diameter, 6 mL for >3 cm and ≤5 cm, and 9 mL for >5 cm) of the agent consisting of 0.5% w/v hydrogen peroxide and 0.83% w/v sodium hyaluronate was injected into the breast tumor twice a week (Monday and Thursday) under ultrasonographic guidance, just prior to each session of radiation therapy. The injection was started immediately prior to the 6th fraction of radiation therapy to avoid possible increased migration of viable tumor cells into microvessels surrounding tumor tissue.

CT examinations for confirming the production and distribution of oxygen generated from the KORTUC radiosensitization agent were performed at approximately three hours following the second or third injection. CT was performed with an Aquilion TSX-101A (Toshiba Co. Ltd., Tokyo, Japan) with the subject in a supine position. The HU values of the low-density regions generated were measured to confirm that the generated regions on CT images contained a large quantity of oxygen.

Two-dimensional (2D) reconstructed breast CT imaging from 5-mm-thick images and 3D- reconstructed breast CT imaging from images of 1-mm-thick images were used to evaluate the production and distribution of oxygen gas. To evaluate the distribution of oxygen, comparison of the position of the tumor and that of the generated oxygen gas was performed using 3D-MIP imaging of CE breast MRI. CE breast MRI was performed with a Signa EXCITE HDx (GE Healthcare, Milwaukee, WI, USA) with the subject in a prone position [19].

## 5. Conclusions

Through this experiment, the method of KORTUC injection under ultrasonographic guidance was seen to be useful, and it was confirmed that production and distribution of oxygen gas generated from KORTUC radiosensitization was visualized on CT imaging.

Tumor hypoxia, which is considered to be the main cause of radioresistance in currently-used Linac radiation therapy for malignant neoplasms, appears to be resolved by intratumoral KORTUC injection under ultrasonographic guidance in terms of confirming oxygen distribution in the target tumor tissue and around the tumor on dedicated CT imaging.

## Figures and Tables

**Table 1:** Summary of the patients’ data.

Case	Age/Gender	TNM	Diseased Site	Oxygen Distribution
1	61/F	T2N1M0	Rt.B	good
2	48/F	T4cN1M0	Lt.C	good
3	70/F	T2N0M0	Lt.CD	good
4	41/F	T2N3M0	Lt.BD	good
5	39/F	T1cN0M0	Lt.C	good
6	38/F	T2N1M0	Lt.AC	good
7	50/F	T2N1M0	Rt.AC	good
8	51/F	T2N0M0	Rt.D	good
9	54/F	T1cN0M0	Lt.A	good
10	56/F	T3N1M0	Lt.C	good

**Table 2:** Hounsfield unit value of each tissue on CT examination.

Tissue	Hounsfield Unit
bone	400~1000
soft tissue	40~80
blood	30~50
water	0
fat	−60~−100
lung	−800~−900
air	−1000
